# Learning Performance Is Influenced by the Social Environment in Cichlid Fishes

**DOI:** 10.1037/cep0000236

**Published:** 2020-09

**Authors:** Emily Stanbrook, Joseph Jodoin, Brett Culbert, Susanne Shultz, Sigal Balshine

**Affiliations:** 1 Department of Earth and Environmental Sciences, University of Manchester; 2 Department of Psychology, Neuroscience, and Behaviour, McMaster University; 3 Department of Earth and Environmental Sciences, University of Manchester; 4 Department of Psychology, Neuroscience, and Behaviour, McMaster University

**Keywords:** comparative learning, cichlids, sociality, Lamprologines, associative and social learning, apprentissage comparatif, cichlidés, sociabilité, Lamprologus, apprentissage associatif et social

## Abstract

It has been hypothesised that some specialised cognitive abilities may have evolved because of the challenges of living in complex social environments. Therefore, more-social species might be able to learn faster than less-social species. The aim of this study was to develop a learning framework to test how more- and less-social Lamprologine cichlid fishes perform across associative learning tasks. These cichlids are a group of closely related species with similar ecologies and life histories but varying degrees of sociality, making them an ideal group for comparative learning studies. We found that three nongrouping cichlids (*Telmatochromis temporalis, Lamprologus meleagris*, and *Neolamprologus tretocephalus*) outperformed three closely related highly social, cooperatively breeding cichlids (*N. pulcher, N. multifasciatus*, and *Julidochromis dickfeldi*) on an associative learning task based on food rewards. However, we hypothesised that these differences may be caused by the social environment during testing and might not reflect true cognitive differences. Indeed, when we drilled down and compared just two species across four different social conditions, we found that the social environment during learning trials affected the performance of the highly social *N. pulcher* and the less-social *T. temporalis* differently. We then performed further experiments with both *N. pulcher* and *T. temporalis* under more natural social settings. Under these more natural social conditions, we found that *N. pulcher* learned to differentiate accessible and inaccessible shelters faster than *T. temporalis*. These findings highlight the potential for expanding comparative experiments investigating the relationship between sociality and cognition and emphasise the crucial role social environment plays in learning outcomes.

## Public Significance Statement

We found that differences in learning speed between more- and less-social fish are dependent on social conditions during testing. Additionally, although less-social fish were faster to learn a novel foraging task compared to more-social fish, the more-social *Neolamprologus pulcher* learned to distinguish accessible and inaccessible shelters faster than the less-social *Telmatochromis temporalis* when in natural social settings. We suggest that social conditions during testing are a critical factor in comparing species’ learning abilities.

## Abstract (fr)

On a émis l’hypothèse selon laquelle certaines capacités cognitives spécialisées pourraient avoir évolué en raison des défis de la vie dans des environnements sociaux complexes. Par conséquent, les espèces plus sociales pourraient apprendre plus rapidement que les espèces moins sociales. L’objectif de cette étude était de mettre au point un cadre d’apprentissage pour tester la façon dont les poissons-cichlidés Lamprologus plus et moins sociaux se comportent dans le cadre de tâches d’apprentissage associatif. Ces cichlidés sont un groupe d’espèces étroitement liées avec des écologies et des historiques de vie similaires, mais avec des degrés variables de sociabilité, ce qui en fait un groupe idéal pour les études d’apprentissage comparatives. Nous avons constaté que trois cichlidés non regroupés (*Telmatochromis temporalis, Lamprologus meleagris*, et *Neolamprologus tretocephalus*) ont surpassé trois cichlidés très sociaux, étroitement liés et se reproduisant en coopération (*N. pulcher, N. multifasciatus et Julidochromis dwickfeldi*) dans une tâche d’apprentissage associatif basée sur les récompenses alimentaires. Cependant, nous avons émis l’hypothèse que ces différences peuvent être causées par l’environnement social pendant les tests et pourraient ne pas refléter de réelles différences cognitives. En effet, lorsque nous avons creusé et comparé seulement deux espèces dans quatre conditions sociales différentes, nous avons constaté que l’environnement social pendant les essais d’apprentissage avait une incidence différente sur le rendement de l’espèce très sociale, *N. pulcher* et de l’espèce moins sociale, *T. temporalis*. Nous avons ensuite réalisé d’autres expériences avec *N. pulcher* et *T. temporalis* dans des milieux sociaux plus naturels. Dans ces conditions sociales plus naturelles, nous avons découvert *que N. pulcher* avait appris à distinguer les abris accessibles et inaccessibles plus rapidement *que T. temporalis*. Ces résultats soulignent le potentiel d’élargir les expériences comparatives en étudiant la relation entre la sociabilité et la cognition et mettent l’accent sur le rôle crucial que joue l’environnement social dans les résultats d’apprentissage.

The ability to learn effectively is crucial for many species’ survival. By accumulating knowledge through personal experience, individuals can better exploit their environment by employing new skills and altering their behaviour in ways that potentially increase survival. For example, individuals can learn to forage more efficiently and better avoid predators (Brown & Laland, 2003; Griffin, Blumstein, & Evans, 2000). Indeed, there are several examples of basic survival skills that do not appear to be innate but instead must be learned. The mortality rate of hatchery-reared fish released into the wild is much greater than for wild fish (Brown & Laland, 2001; Rodewald, Hyvärinen, & Hirvonen, 2011), and similarly, translocated or reintroduced animals are often more likely to be predated (Griffin et al., 2000; Shier & Owings, 2007). In both instances, mortality can be reduced by training individuals to respond more appropriately to anticipated challenges.

However, not all species are equally capable of learning, with the most sophisticated learning abilities documented in primates (particularly apes, capuchins, and some Old World monkeys), several bird groups (such as parrots and crows), and toothed cetaceans (MacLean et al., 2014; Marino et al., 2007; Roth & Dicke, 2005; van Horik, Clayton, & Emery, 2012). That these groups are also generally considered to be highly socially complex has led to several hypotheses linking sociality to cognitive ability (Clutton-Brock & Harvey, 1980; Dunbar, 1998; Dunbar & Shultz, 2007; Fox, Muthukrishna, & Shultz, 2017; Jerison, 1963; Whiten & Byrne, 1988). In particular, the social brain hypothesis posits that greater cognitive ability has evolved in species with greater sociality because sociality is inherently cognitively challenging (Dunbar, 1998). For example, social individuals may need to resolve conflicts with, coordinate movements with, and even simply recognise group members, whereas solitary individuals do not (Dunbar & Shultz, 2007). Hence, it is possible that some aspects of cognitive ability are coupled with sociality. Although most species can learn about their environment, more-social species should be better able to learn than less-social species (Klopfer, 1959; Leadbeater, 2015; van Schaik & Burkart, 2011). This may be particularly true for explicitly social tasks. Evidence for the social brain hypothesis mostly comes from studies that have correlated brain size, especially neocortex size, with the degree of sociality (Dunbar, 1992; Finlay & Darlington, 1995; Joffe & Dunbar, 1997; Pawłowski, Lowen, & Dunbar, 1998). In turn, several studies have correlated brain size and problem-solving ability (Benson-Amram, Dantzer, Stricker, Swanson, & Holekamp, 2016; Deaner, Isler, Burkart, & van Schaik, 2007; Lodato & Arlotta, 2015; Lui, Hansen, & Kriegstein, 2011; MacLean et al., 2014; Sakai, Arsznov, Hristova, Yoon, & Lundrigan, 2016; Shultz & Dunbar, 2010).

An alternative to the hypothesis that sociality is predictive of learning ability is the idea that more complex social environments are more information rich than simpler social environments (Danchin, Giraldeau, Valone, & Wagner, 2004; Muthukrishna & Henrich, 2016). Muthukrishna and Henrich (2016) suggested that social living allows for the emergence of a “collective brain” whereby the social network acts as an information transfer network, allowing groups to pool their cognitive resources. Essentially, more-social species may learn better than less-social species simply because their social structure presents them with more opportunities to exploit easily accessible information (Muthukrishna & Henrich, 2016). For example, a solitary individual attempting to access a novel food source can only learn through trial and error, while a social species can acquire information about successful strategies from its group mates.

Whether more-social species are better able to learn than less-social species and whether this is due to fundamental cognitive differences or dependent on the social network an individual is in is largely untested. Testing these hypotheses requires a comparative learning paradigm that can account for social environment and ecological differences between species. Hence, here we aimed to establish such learning paradigms to test whether more-social species are innately better able to acquire and use information than less-social species or whether species differences are mediated by different social environments using Tanganyikan cichlid fishes.

Cichlid fishes are an ideal model system to test how cognition differs between more-social and less-social species (Pollen et al., 2007). Cichlids are a highly speciose group that displays a range of social behaviours, with all species engaging in some form of parental care (Duponchelle, Paradis, Ribbink, & Turner, 2008; Whiteman & Côte, 2004). One group of closely related cichlids, the Lamprologines, endemic to Lake Tanganyika, are especially useful models because many of these species have very similar ecologies and life histories yet are particularly socially diverse (Dey et al., 2017; Pollen et al., 2007). Some species of this group are mostly solitary, while others live in pairs, in colonies, or even in obligate cooperatively breeding groups (Axelrod, 1993; Barlow, 1991; Brichard, 1989). In this study, we focused mainly on two closely related Lamprologine cichlids: *Neolamprologus pulcher* and *Telmatochromis temporalis*. *N. pulcher* is a highly social, obligate cooperative breeder that lives in groups of up to 20 (Wong & Balshine, 2011), whereas *T. temporalis* is more solitary, sometimes found living in breeding pairs, but does not tolerate other conspecifics in its territories (O’Connor, Marsh-Rollo, Aubin-Horth, & Balshine, 2016). We also expanded our investigations of comparative cognition by testing two more cooperatively breeding group-living cichlids, *N. multifasciatus* and *Julidochromis dickfeldi*, and their most closely related more solitary relatives, *Lamprologus meleagris* and *N. tretocephalus*. We chose these species to be representative of the range of social behaviours and morphologies found across Lamprologine cichlids to ensure, as far as possible, that the developed protocol could be used in other species in the future. All of the species included maintain similar territories and have similar diets; however, *N. pulcher* feed primarily on midwater zooplankton, whereas *T. temporalis* typically scrape algae from rocks (aufwuchs), *N. tretocephalus* and *L. meleagris* feed on zoobenthos, *N. mulifasciatus* feed on zoobenthos and midwater zooplankton, and *J. dickfeldi* are omnivorous (Hori, Yamaoka, & Kenzi, 1983; Konings, 1998).

In all, we conducted a series of four experiments using either all six species (Experiment 1) or only two species (*N. pulcher* and *T. temporalis*; Experiments 2–4). In Experiments 1 and 2, we tested individuals’ abilities to learn to access a hidden food reward on a food tray. We used a common paradigm to test associative learning (Buechel, Boussard, Kotrschal, van Der Bijl, & Kolm, 2018; Lucon-Xiccato & Bisazza, 2014), making these results comparable with other experimental findings and future experiments using this approach. In Experiment 2, we tested how single focal fish learned about food rewards under different social conditions to ascertain how the presence of a conspecific impacted learning in *N. pulcher* and *T. temporalis*. In Experiments 3 and 4, we tested individuals’ abilities to learn about shelter. We expected shelter to be a particularly ecologically salient reward for both species because they both use shelters for predator avoidance and breeding under natural social conditions (Brown & Chivers, 2005; Coleman & Rosenthal, 2006; O’Connor, Reddon, Odetunde, Jindal, & Balshine, 2015). Overall, we tested two competing hypotheses. First, if sociality drives the evolution of cognitive ability, then the more-social *N. pulcher* (and *N. multifasciatus* and *J. dickfeldi*) should outperform the less-social *T. temporalis* (and *L. meleagris* and *N. tretocephalus*) in every learning task. Second, if the social environment provides more opportunities for learning (Muthukrishna & Henrich, 2016), then the more- and less-social species should perform similarly when held under identical social conditions, as in Experiments 1 and 2, and the more-social species would only outperform the less-social species when held in natural social conditions, as in Experiments 3 and 4.

## Method and Results

### General Housing, Behavioural Assays, and Statistical Analyses

Experiments were conducted from May to September in 2016 and 2017 using sexually mature fish held at the tropical breeding facility at McMaster University. Fish were laboratory- or aquarium-reared descendants of wild-caught fish from Lake Tanganyika, Africa. Experimental fish were held in either 450L or 150L stock tanks that were maintained at 26 ± 2 °C, with a 13L:11D photoperiod. Fish were fed dried cichlid food ad libitum six times per week.

Every trial was video recorded using a Cannon Vixia HF S100, and all data extracted from videos were scored by a single observer within a single experiment (Emily Stanbrook or Joseph Jodoin). Statistical analyses were conducted using R (Version 3.6.1; R Core Team, 2019). All data are presented as either proportions or means ± 1 standard error of the mean (SEM), and a significance level of 0.05 was used for all tests. When data did not meet the assumptions of normality and/or equal variance, data were log transformed; if the data could not be transformed to meet these assumptions, then equivalent nonparametric analyses were performed. Cox proportional hazard (PH) models were fit using the coxme package (Therneau, 2020a), survival curve differences were tested using the survival package (Therneau, 2020b), and Tukey’s honest significant difference (HSD) tests were performed using the emmeans package (Lenth, Singmann, Love, Buerkner, & Herve, 2019).

### Ethical Statement

The experimental protocols used in all experiments were approved by McMaster University’s Animal Research Ethics Board (Animal Utilization Protocol 14–02-05) and adhere to the guidelines of the Canadian Council for Animal Care. The experimental protocols used in Experiments 1 and 2 were also approved by the University of Manchester animal ethics committee and adhere to U.K. Home Office regulations. We carefully monitored the condition of every fish daily and minimised handling and stress. Two *T. temporalis* experienced minor injury (scale loss and torn fins) during Experiment 3 trials and were immediately removed and treated. For Experiments 1 and 2, all fish that failed to feed for the 4-day training phase were returned to their stock tanks and observed to feed (dried cichlid food) within 1 day of their return.

## Experiment 1: Do More-Social Cichlids Learn to Access Food Faster Than Less-Social Cichlids?

To develop a simple paradigm to compare species performance on an associative task, we modified a protocol designed for guppies by Lucon-Xiccato and Bisazza (2014) and developed further by Buechel et al. (2018) that requires fish to move disks to access a food reward hidden underneath. We simplified the protocol such that we only tested associative learning (by operant conditioning) and removed the likely more cognitively demanding reversal learning component (Güntürkün, 2005; Izquierdo, Brigman, Radke, Rudebeck, & Holmes, 2017). To ensure that this paradigm could be used across multiple cichlid species, we trained three cooperatively breeding/more-social cichlid species (*N. pulcher*, *N. multifasciatus*, and *J. dickfeldi*) and three closely related noncooperatively breeding/less-social cichlid species (*T. temporalis*, *L. meleagris*, and *N. tretocephalus*) on this task.

### Method

**Figure 1 fig1:**
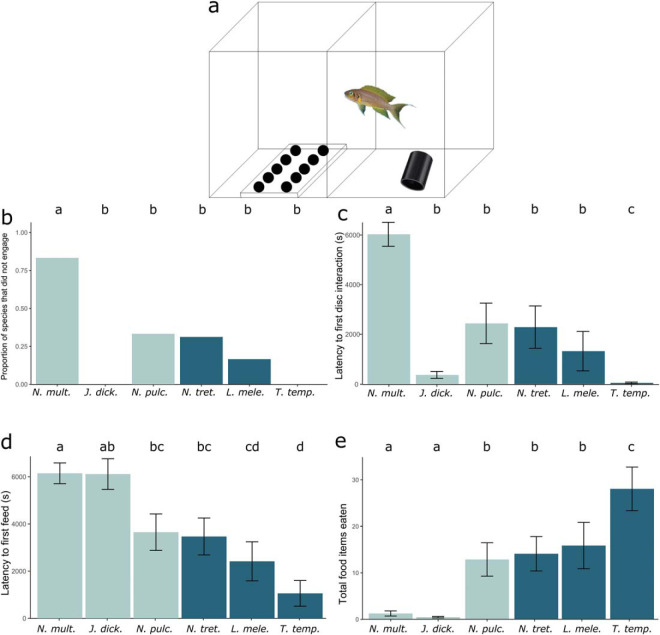
Experiment 1. Panel a: An illustration of the aquaria set up for the cross-species feeding experiment. Panel b: The proportion of fish of each species that never engaged with the feeding apparatus. Panel c: The mean latency to first disk interaction by each species. Panel d: The mean latency (in seconds) taken by each species to uncover fully hidden food. Panel e: The mean number of food items accessed by each species. Species that share a letter do not have statistically significantly different latencies. Error bars show standard error of the mean. Cooperatively breeding, group-living, more-social species are in light blue (light gray; in order: *N. multifasciatus, J. dickfeldi,* and *N. pulcher*). Noncooperatively breeding, less-social species are in dark blue (dark gray; in order: *N. tretocephalus, L. meleagris,* and *T. temporalis*).

All fish were first housed singly in 10L aquaria with an air stone, heater, PVC tube as a shelter (9.4 × 5.2 cm), and 3 cm of coral sand as substrate (Figure 1a) for an initial overnight acclimation, followed by 7 days of training and trials in these same aquaria. During the training and trials, opaque white acrylic feeding trays, 20 × 10 × 1 cm for the larger species and 10 × 5 × 1 cm for the considerably smaller *N. multifasciatus* and *L. meleagris*, were placed in the front half of each aquarium. Each feeding tray had 10 wells (0.5 cm deep and either 2 cm or 1 cm in diameter corresponding to the size of the tray). While the feeding tray was being placed in the testing chamber of the aquaria, the view of the test fish into the test chamber was entirely obstructed by an opaque barrier. Each well of the feeding tray was loaded with a bloodworm (for the four larger species) or brine shrimp (for the two smaller species). Ten black acrylic disks (larger disks = 3 cm diameter, 0.3 cm thick, 2.3 g; smaller disks = 1.5 cm diameter, 0.2 cm thick, 0.5 g) were placed on top of the trays and with the disks increasingly covering the food wells with the worms or shrimp as trials progressed (Figure 1a). Fish were trained over eight sessions. In Sessions 1–2, the disks were placed beside the food wells, but the worms or brine shrimp in the wells were completely visible. In Sessions 3–4, the disks covered one third of the well, while in Sessions 5–8, the disks covered two thirds of the well. Fish that never fed from any wells during training were considered to be training dropouts because they never engaged with the experimental apparatus (see results, Figure 1b). All dropouts were excluded from the experiments, returned to stock aquaria, and monitored to ensure they fed immediately. Fish that reached the trial phase went through six trials where the disks completely covered the food wells (termed the test phase). All 10 wells had a food reward. Fish simply had to learn to remove the disk to reach the food.[Fig-anchor fig1]

After the filled feeding tray and disks had been placed in the aquaria, the opaque barrier was removed, and the fish was free to interact with the feeding tray for 20 min, its activity was filmed, and latency to first disk interaction (touch) and first feed as well as the total number of accessed wells was recorded. Following this 20-min test period, the fish was gently guided back into its holding area, the opaque barrier replaced, and the feeding apparatus removed. To maintain the motivation to feed, each fish completed a maximum of two trials per day with a minimum time of 4 hr and a maximum time of 6 hr between trials.

### Statistical Analysis

We used a linear model to test whether dropout rates were different between species. To test whether there were species differences in performance in the task, we assessed latency to interact with the disks for the first time and latency to uncover the food for the first time by fitting Cox PH models and assessed the total number of food items accessed by fitting a generalised linear model (GLM) with a Poisson distribution. All models used species and sociality (more- or less-social) as fixed effects. As dropouts did not complete the test phase, they were included in each model with their response variable set either to the maximum (latency to uncover food and latency to first disk interaction) or minimum value (total number of food items eaten). Either survival curves were compared using the log-rank test or a post hoc Tukey’s HSD test was performed to assess pairwise species’ differences. We could then determine whether the more-social species (*N. pulcher, N. multifasciatus,* and *J. dickfeldi*) consistently performed better in any of these measures than the less-social species (*T. temporalis, L. meleagris,* and *N. tretocephalus*).

### Results

Overall, less-social species performed better in this foraging-based learning task than the more-social species (Figure 1b−e). *N. multifasciatus* were least likely to engage with the task (*p* < .001). Only 17% of the *N. multifasciatus* (5/30) advanced through training to the trial phase. In contrast, 66% of the *N. pulcher* (12/18), 100% of the *J. dickfeldi* (11/11), 100% of the *T. temporalis* (12/12), 83% of the *L. meleagris* (10/12), and 69% of the *N. tretocephalus* (11/16) completed training (Figure 1b).

Performance varied between species (Cox PH: χ^2^ = 30.0, *df* = 4, *p* < .001), and social species had longer latencies to interact with the disks (Cox PH: χ^2^ = 25.5, *df* = 1, *p* < .001). *N. multifasciatus* were the slowest to interact with the disks, while *T. temporalis* were the fastest, and all other species performed comparably (Figure 1c). Less-social species were also faster to uncover food for the first time during trials (Cox PH: χ^2^ = 25.4, *df* = 1, *p* < .001), and there was also variation between species (Cox PH: χ^2^ = 18.5, *df* = 4, *p* = .001). *N. multifasciatus* and *J. dickfeldi* took the longest to uncover fully hidden food of all six species, with *T. temporalis* and *L. meleagris* uncovering food the fastest (Figure 1d). Species (GLM: *F* = 108, *df* = 4, *p* < .001) and sociality (GLM: *F* = 462, *df* = 1, *p* < .001) were also highly predictive of how many food items were accessed. As predicted, the relationship between species and amount of food eaten was largely the inverse of the relationship between species and latency to uncover food; that is, species that were faster to uncover food also ate more food items (Figure 1e). *N. multifasciatus* and *J. dickfeldi* ate significantly fewer food items than all other species, and *T. temporalis* ate the most food items.

## Experiment 2: Does Social Environment Differently Impact How Fast More-Social *N. Pulcher* and Less-Social *T. Temporalis* Learn to Access Food?

In Experiment 1, we tested more- and less-social fish under isolation. We hypothesised that the generally poor performance of the more-social fishes may have been disproportionately negatively impacted by the single fish housing conditions during testing, which represents an unnatural state for these highly social fish species. The possible interaction between housing and sociality might also account for the higher dropout rate and poor performance in the more-social *N. multifasciatus* and *J. dickfeldi*, respectively. Although there is widespread evidence that cognitive performance in social species is negatively affected by social isolation (Cacioppo & Hawkley, 2009), how social environments might differentially impact learning ability in more- and less-social species has not been widely addressed. Therefore, in this second experiment, we repeated Experiment 1 under three additional social conditions to explore the role of social environment in task performance in more-social *N. pulcher* and less-social *T. temporalis*. We predicted that the more-social *N. pulcher* would perform better under more social conditions, whereas the less-social *T. temporalis* would perform worse with extra social stimuli.

### Method

**Figure 2 fig2:**
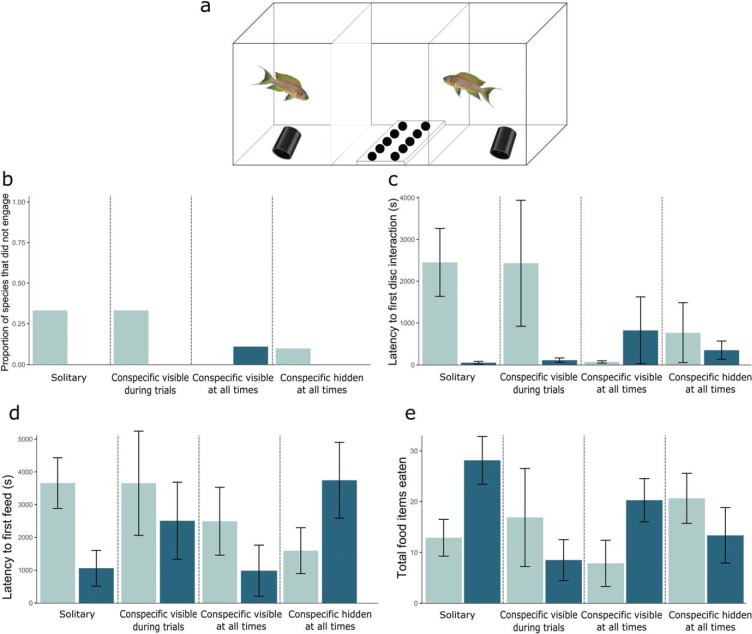
Experiment 2. Panel a: The aquarium set up for Experiment 2. Panel b: The proportion of fish of each species that never engaged with the test apparatus. Panel c: The mean latency to first disk interaction by each species. Panel d: The mean latency (seconds) of each species to uncover fully hidden food over the four social conditions of solitary, conspecific visible during trials, conspecific always visible, and social control of a conspecific always present but hidden. Panel e: The mean number of food items accessed by each species. Error bars show standard error of the mean. Cooperatively breeding species are in light blue (light gray); noncooperatively breeding species are in dark blue (dark gray).

As in Experiment 1, *N. pulcher* and *T. temporalis* were trained to access food hidden under black acrylic disks, with all disks hiding a food reward. However, the fish were exposed to three additional social conditions during training and trials. In the first condition, the test fish could always see a conspecific during the entire 7 days of training and testing, that is, prior to, during, and following each trial (*n* = 10 *N. pulcher*, *n* = 9 *T. temporalis*). In the second condition, the test fish could see a conspecific only during the 20-min feeding trials, while for the rest of the time, the conspecific was hidden behind an opaque barrier (*n* = 6 *N. pulcher*, *n* = 9 *T. temporalis*). In the third condition, a conspecific was always present but was hidden from the test fish behind an opaque barrier (*n* = 10 *N. pulcher*, *n* = 10 *T. temporalis*). Fish that never fed from any wells during training were again removed from the test aquaria and did not undergo trials. Conspecifics were sex and size matched to the focal fish (within 5% standard length of the focal fish), and these trials took place in 30L aquaria to accommodate the conspecific social stimuli fish. The test aquaria were separated into thirds, with the focal test fish in one end, a neutral central third where the feeding tray was set up, and a final third of the aquaria that housed the size- and sex-matched conspecific (Figure 2a). Barriers were either opaque or clear, depending on social condition. Otherwise, housing conditions were identical to those in Experiment 1.[Fig-anchor fig2]

### Statistical Analysis

Although focal fish in the third social condition (conspecific always present but hidden from the focal fish) were never able to interact directly with a conspecific, because the barriers between fish were not fully water tight, it is likely that they could perceive the presence of a conspecific through auditory or chemical cues. Therefore, to compare all social conditions and to control for any potential effects of these cues, we included the truly solitary/nonsocial condition *N. pulcher* and *T. temporalis* data from Experiment 1 in the analyses and figures. Hence, we compared performance in feeding trials across four social conditions: under the truly solitary holding conditions in Experiment 1 (focal fish never saw, smelled, or heard conspecifics), under an intermediate social condition (focal fish saw a conspecific during feeding trials only), under an always social condition (focal fish saw a conspecific at all times), and under a social control condition (a conspecific was always present but behind a barrier so could not be seen by the focal fish). We compared task performance by fitting Cox PH models to compare latencies to interact with the disks and uncover food using the interaction between species and social condition interaction as a fixed effect. We also fit a GLM to test whether *N. pulcher* accessed more food than *T. temporalis* using the same variable as above. As in Experiment 1, dropouts were included in each model with their response variable set either to the maximum (latency to uncover food and latency to first disk interaction) or minimum value (total number of food items eaten).

### Results

In general, the less-social *T. temporalis* performed better than the more-social *N. pulcher* under both the truly solitary and always social (conspecific visible at all times) conditions but performed comparably to *N. pulcher* in the intermediate condition and worse than *N. pulcher* in the social control condition (Figure 2b−e). As with Experiment 1, there were species differences in engagement with the task. In particular, fewer *N. pulcher* completed the task in the intermediate social condition (focal fish saw a conspecific during feeding trials only; Figure 2b). *N. pulcher* completed 67% (12/18) of the training trials in the solitary condition, 67% (4/6) in the intermediate social, 100% (10/10) in the constantly social condition, and 90% (9/10) in the social control condition 90% (9/10). For the *T. temporalis*, 100% completed the training trials in the solitary (12/12), intermediate (9/9), and social control (10/10) conditions, and 89% (8/9) in the most social condition.

Species (Cox PH: χ^2^ = 4.62, *df* = 1, *p* = .032) interacted with social condition (Cox PH: χ^2^ = 12.5, *df* = 3, *p* = .006) to influence the latency to interact with the disks (Figure 2c). Latency to uncover food was not affected by species, social condition, or the interaction between both variables (Figure 2d). The total number of food items eaten was affected by species (GLM: χ^2^ = 15.8, *df* = 1, *p* < .001), social condition (GLM: χ^2^ = 64.9, *df* = 3, *p* < .001), and the interaction between species and social condition (GLM: χ^2^ = 146, *df* = 3, *p* < .001; Figure 2e).

## Experiments 3 and 4: Can *N. Pulcher* Distinguish Between Accessible and Inaccessible Shelters Faster Than *T. Temporalis*?

Surprisingly and contrary to our initial prediction, overall, the less-social *T. temporalis* generally outperformed the more-social *N. pulcher* in both Experiments 1 and 2. In Experiments 3 and 4, we used a shelter reward on the assumption that in the predator-rich waters of Lake Tanganyika (Sarvala, Tarvainen, Salonen, & Mölsä, 2002) where food for *N. pulcher* and *T. temporalis* is abundant and not easily defended (Balshine et al., 2001; Mboko, Kohda, & Hori, 1998), shelter represents a more salient and comparable reward for both species. We tested whether *N. pulcher* would be slower than *T. temporalis* to learn about a refuge. Further, as we found that social environment greatly influenced the learning ability of both species, in this experiment, we kept both species in their natural states during testing. Therefore, *N. pulcher* were held in cooperatively breeding groups, and *T. temporalis* were held in breeding pairs. Together, these changes allowed us to test learning under more natural conditions.

### Method

**Figure 3 fig3:**
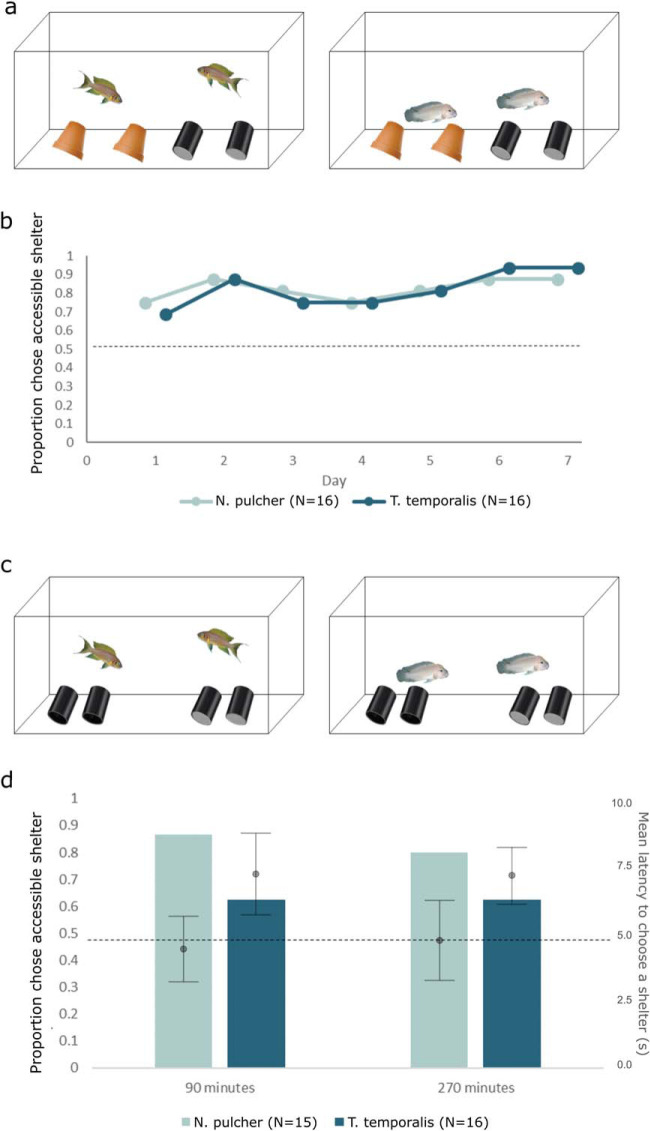
Experiments 3 and 4. The shelter configurations for Experiments 3 (Panel a) and 4 (Panel c). Half the shelters were made inaccessible by sealing with clear acrylic. Panel b, Experiment 3: The proportion of fish that chose the accessible shelters across 7 days of testing (one startle per day). Panel d, Experiment 4: The proportion of fish (bars) that chose the accessible shelters following startles at 90 and 270 min and the mean latency (circles) of each species to choose a shelter. The more-social, cooperatively breeding *N. pulcher* is shown in light blue (light gray), and the less-social, noncooperatively breeding *T. temporalis* is in dark blue (dark gray).

In Experiment 3, 16 *N. pulcher* breeders (i.e., eight pairs) and 18 *T. temporalis* (i.e., nine pairs) breeders were used as focal fish. *N. pulcher* were kept in groups with a breeding pair and several helpers (mean number of helpers = 2.5, SEM = 0.19). Fish were fin clipped for individual identification, with no adverse behavioural effect (Stiver, Dierkes, Taborsky, & Balshine, 2004). Each group or pair was housed for the duration of testing in a 189L aquaria with a heater, two sponge filters and 3 cm of coral sand, and four shelters (two black PVC tubes [9.4 × 5.2 cm] and two terracotta flowerpot halves [9.4 × 5.2 cm]). In half of the trials, two tube shelters were placed on the left of the aquaria, and two pot shelters were placed on the right side; in the other half of the trials, the sides were switched. Half of the focal fish experienced accessible tubes (where fish could swim in), while the pots were inaccessible (sealed with clear acrylic; see Figure 3a). In the other half of the trials, pots were accessible, and tubes were inaccessible. Hence, the positions of the shelters within the aquaria and the type of shelter that was accessible were counterbalanced across the species and trials. Each species was free to interact with the shelters for 24 hr prior to testing. Prior to testing, the aquaria sponge filters were removed so that the only places to hide were the experimental shelters. Following a 5-min acclimation, fish were startled by moving a net (20 × 15 cm) in a figure-eight motion three times at midwater (average duration of 6.3 s ± 0.07 s). Startles occurred once per day for 7 days between 12:00 and 16:00 to control for diurnal effects, and the behaviour of the fish was recorded for 5 min on video.[Fig-anchor fig3]

In Experiment 4, we essentially repeated Experiment 3 but with increased difficulty in two ways. First, we used only tube shelters so that fish could only distinguish accessible and inaccessible shelters by location, that is, not by location and type of shelter. Second, we ran the trials over a shorter time period (5 hr rather than 7 days) to explore whether both species would learn to distinguish accessible from inaccessible shelters to the same degree of accuracy if given less exposure time with the new shelters. In this fourth experiment, we used 16 naive *N. pulcher* breeders held in groups (mean number of helpers = 2.4, SEM = 0.26) and 18 naive *T. temporalis* held as breeding pairs kept in the same conditions as described above for Experiment 3. However, in this experiment, the fish were not given any experimental shelters until after their minimum 24-hr acclimation period, and only tubes were used as shelter (two accessible tubes placed on one side of the tank and two inaccessible tubes placed on the opposite side in a counterbalanced alternating design; Figure 3c). Before each trial, sponge filters were removed. At 90 and 270 min after the four tubes were placed in the aquaria, the fish were startled as described in Experiment 3. We recorded the response to each startle for 30 min using a video camera and then scored the behaviours from the video recordings.

### Statistical Analysis

In both Experiment 3 and 4, we used binomial tests to determine whether individual *N. pulcher* or *T. temporalis* chose accessible over inaccessible shelters more often than chance (50%). To investigate differences in shelter choice and latency to choose a shelter between species and across trials, we fit generalised linear mixed models (GLMMs; binomial). In Experiment 3, for both models, fixed effects were species, sex, starting distance (number of focal fish body lengths) from accessible shelter, trial number, and shelter type (pot vs. tube); aquarium number was a random effect. In Experiment 4, the side of the tank the accessible shelters were on, instead of shelter type, was included as a fixed effect. In both experiments, all fish engaged with the task, with all fish swimming toward shelter following a startle in at least one trial.

### Results

In Experiment 3, both species chose accessible shelters more often than the inaccessible shelters (80% of cases, binomial test: probability of success = 0.82, 95% CI [0.74, 0.89], *n* = 112, *p* < .001; Figure 3b). There was no evidence that *N. pulcher* or *T. temporalis* performed better or worse in this task (GLMM: χ^2^ = 0.67, *df* = 1, *p* = .412) or even that the fish improved over the trial days (GLMM: χ^2^ = 0.57, *df* = 1, *p* = .450). Both species moved to the “correct side” 80% of the time and remained at this level over the entire experiment. Males were more likely to choose accessible shelters than were females (GLMM: χ^2^ = 8.8, *df* = 1, *p* = .003), and fish that were closer to the accessible shelters at the beginning of the trials were more likely to choose the accessible shelters (GLMM: χ^2^ = 44, *p* < .001) and were faster at doing so (GLMM: χ^2^ = 37, *df* = 1, *p* < .001). We detected no clear difference in either species’ latency to choose accessible shelter (Figure 3b; GLMM: χ^2^ = 0.63, *df* = 1, *p* = .4285) or in their improvement over trials (GLMM: χ^2^ = 0.50, *df* = 1, *p* = .478).

In Experiment 4, after both the first (90 min) and second (270 min) startles, *N. pulcher* chose accessible shelters significantly more often than chance (binomial test: startle 1 probability of success = 0.87, 95% CI [0.60, 0.98], *n* = 15, *p* = .007; startle 2 probability of success = 0.87, [0.52, 0.96], *n* = 15, *p* = .035; Figure 3d). Conversely, *T. temporalis* did not choose accessible shelters more often than by chance after either the first or second startle (both startle responses were the same, binomial test: probability of success = 0.63, [0.35, 0.85], *n* = 16, *p* = .456). *N. pulcher* were also faster to choose accessible shelters than *T. temporalis* (mean latency [seconds]: startle 1 *N. pulcher* = 4.7, *T. temporalis* = 7.4; startle 2 *N. pulcher* = 5, *T. temporalis* = 6.9; GLMM: χ^2^ = 30, *df* = 1, *p* < .001). As in Experiment 3, fish that were closer to the accessible shelters at the beginning of the trials were faster to choose the accessible shelters in both species (GLMM: χ^2^ = 89, *df* = 1, *p* < .001).

## Discussion

Interest in comparative cognition has greatly increased in recent years, and the field is developing rapidly (Heyes, 2017; Whiten, 2017; Whiten, Caldwell, & Mesoudi, 2016). Several studies have linked sociality to brain size (Dunbar, 1992; Finlay & Darlington, 1995; Joffe & Dunbar, 1997; Pawłowski et al., 1998) and brain size to learning ability (Benson-Amram et al., 2016; Deaner et al., 2007; Lodato & Arlotta, 2015; Lui et al., 2011; MacLean et al., 2014; Sakai et al., 2016; Shultz & Dunbar, 2010), yet there is still limited direct research into the relationship between sociality and learning (Byrne & Bates, 2007; Hoppitt & Laland, 2013). We explored two conflicting hypotheses. The first was that if sociality drives the evolution of cognitive ability (Dunbar, 1998; Dunbar & Shultz, 2007), then more-social species should perform better on all learning tasks. The second was that if a more-social environment provides more opportunity for learning (Muthukrishna & Henrich, 2016), then more-social species should perform better only when in natural social groups. To this end, we assessed whether sociality is a reliable predictor of associative learning ability in cichlid fishes across four experiments. Contrary to our predictions, in Experiment 1, we found evidence that less-social species outperformed more-social species in a foraging task; however, in Experiment 2, we found that the social environment during the foraging task significantly affected performance in both more- and less-social species. Specifically, *T. temporalis* outperformed *N. pulcher* in the truly solitary and most-social (conspecific always visible) conditions, whereas both species performed comparably in the intermediate social condition (conspecific visible only during trials) and *N. pulcher* outperformed *T. temporalis* in the social control condition (conspecific present but always invisible). Furthermore, Experiments 3 and 4 together suggest that the more-social *N. pulcher* learns faster than the less-social *T. temporalis* in a shelter use task. Specifically, under more natural social conditions, *N. pulcher* consistently learned to distinguish between accessible and inaccessible shelter more quickly (within 90 min; Experiment 4) than *T. temporalis* (more than 4.5 hr; Experiment 3).

Such differences in learning ability in closely related cichlids are unsurprising because although total brain mass does not appear to be associated with sociality (Reddon et al., 2016), region-specific size differences across cichlid species have been reported (Gonzalez-Voyer & Kolm, 2010b). In other species, telencephalon and cerebellum volume, brain regions associated with higher-order cognition, such as learning (Rodríguez et al., 2005; Salas, Broglio, & Rodríguez, 2003), have been linked with both habitat complexity and indicators of sociality such as mating system (Gonzalez-Voyer & Kolm, 2010a; Pollen et al., 2007). Conversely, the results from our foraging experiments (Experiments 1 and 2) did not support the hypothesis that more-social species have greater cognitive abilities as the more-social species did not consistently learn to find food faster than the less-social species. In particular, the more-social *N. pulcher* were either slower or equally slow at finding hidden food compared to the less-social *T. temporalis* regardless of the social environment.

Although we used two different food rewards in Experiment 1 (blood worms or brine shrimp) to ensure that all species could feed irrespective of their body size, both rewards used are small invertebrates, similar to those readily consumed by these species in the wild (Hata, Shibata, Omori, Kohda, & Hori, 2015; Wagner, McIntyre, Buels, Gilbert, & Michel, 2009). Therefore, it is unlikely that reward differences account for the results of Experiment 1. However, as the design of Experiments 1 and 2 required individuals to feed from an apparatus near the bottom of the aquaria, this may have favoured *T. temporalis* as this species primarily eats algae (aufwuchs) from rock surfaces (Takamura, 1984), whereas *N. pulcher* more commonly feed on zooplankton from the water column (M. Taborsky, 1984). Conversely, *N. tretocephalus* is also a benthic feeder (Axelrod, 1993; Brichard, 1989), yet *N. tretocephalus* performed similarly to *N. pulcher*, indicating that differences in feeding habits were likely not major determinants of performance during this food-based learning task. However, the different proportions of fish of each species that failed to feed (dropouts) even when the food was not fully hidden in Experiments 1 and 2 may indicate that some species were consistently more stressed by the experimental conditions than others. Hence, each species’ ability to perform the foraging task was potentially differently affected by the configuration of the task itself, thus constraining our ability to directly compare performance between each species. Stress experienced during, but out of context with, a learning event can impair memory (Joëls, Pu, Wiegert, Oitzl, & Krugers, 2006). This was the rationale behind extending the solitary condition of Experiment 1 into the multiple social conditions of Experiments 1 and 2 combined. Indeed, we found that social condition impacts foraging task performance for both *N. pulcher* and *T. temporalis*, although each species’ performance was affected differently across social conditions.

To account for these differential social effects in the two species, Experiments 3 and 4 involved maintaining natural social groups of *N. pulcher* and pairs of *T. temporalis*. In the wild, *N. pulcher* share and likely compete for shelters with their group mates, which may make learning about shelters a more salient task for them than *T. temporalis*. Additionally, because they were held in pairs, *T. temporalis* had one accessible shelter for each fish per aquarium, whereas the *N. pulcher* that were held in groups had one accessible shelter between an average of 2.2 fish. Both factors could drive *N. pulcher* to learn about shelter quality more quickly. This was the motivation for focusing on the learning of only the breeding pair in both species; as *N. pulcher* breeders are the most dominant members of the group, competition for shelters should mainly affect the helpers (B. Taborsky, Arnold, Junker, & Tschopp, 2012). In Experiments 3 and 4, *N. pulcher* potentially had greater exposure to social transmission and reinforcement between individuals. The *N. pulcher* in social groups could observe multiple conspecifics interacting with the experimental apparatus, whereas the *T. temporalis* in Experiments 3 and 4 could observe only one conspecific interacting with the experimental apparatus. Social transmission has been observed in several unrelated grouping and nongrouping fishes (Laland & Williams, 1998; Suboski et al., 1990; Webster & Laland, 2017), demonstrating that many fishes likely have the ability to learn from conspecifics. However, behavioural cues from group mates may be more salient for *N. pulcher,* which live in large cooperatively breeding groups (Wong & Balshine, 2011), than for *T. temporalis,* which commonly live as a breeding pair (O’Connor et al., 2016). Hence, the more-social *N. pulcher* may be more attuned to the social environment and socially available information than the less-social *T. temporalis*, which may explain why *N. pulcher* outperformed *T. temporalis* when held in natural social conditions (Experiments 3 and 4), whereas *N. pulcher* did not generally outperform *T. temporalis* when both species were held in identical social conditions. These results together suggest that differences in learning ability between more- and less-social species are not due to specific adaptations for learning in more-social species but may be due to the increased availability of information within a social group (Heyes, 2012; Lefebvre, Palameta, & Hatch, 1996).

The ability to learn from others is known to be influenced by social rank in hens (*Gallus gallus domesticus*) and dogs (*Canis lupus familiaris*; Nicol & Pope, 1994; Pongrácz, Vida, Bánhegyi, & Miklósi, 2008). While the potential interaction between dominance and learning was one of the motivating factors behind our focus on dominant breeders in Experiments 3 and 4, the housing conditions in Experiments 1 and 2 limited focal individuals’ opportunities to interact with conspecifics and develop hierarchies. Therefore, an alternative explanation of these results could be that the more-social focal fish were more motivated to find conspecifics than to engage with and learn the foraging task, thereby reducing their performance in Experiments 1 and 2. In other words, the inability to interact with conspecifics (rather than a deficit of social information) may have resulted in less-social species outperforming more-social species in Experiment 1 and the lack of a clear difference between *N. pulcher* and *T. temporalis* in Experiment 2. That the truly solitary environment limited the more-social species’ performance is supported by the difference in dropout rates between social conditions; in Experiment 2, *N. pulcher* were least likely to complete the solitary and intermediate social conditions, indicating stress or distraction from being solitary or being around an unfamiliar conspecific. Conversely, the most social condition, a conspecific always present, was the only social condition in which no *N. pulcher*, but some *T. temporalis*, failed to complete the trials. Presumably, a social relationship would have been able to be formed across a barrier when two individuals shared the space for so many days. However, direct physical interaction between focal and conspecific fish in Experiment 2 was not allowed because the close size and sex matching would promote potentially deadly aggression in the pair. For future comparative learning studies, the two aspects of being in a group, reduced stress due to social buffering (for group-living species; Culbert, Gilmour, & Balshine, 2019) and the ability to acquire information efficiently, should be decoupled.

## Conclusion

We found that more-social cichlids do not learn to find food faster than less-social cichlids and that *N. pulcher*, one highly social cichlid, was no better than *T. temporalis*, a less-social cichlid, at finding food across four social contexts. Conversely, we found that *N. pulcher* learned about shelter quality faster than *T. temporalis*. Overall, we found no evidence that more-social cichlids consistently perform better at these two learning tasks than less-social cichlids; therefore, these results do not support the hypothesis that sociality drives the evolution of cognitive ability. Conversely, our results do support the hypothesis that a more-social environment provides more opportunities for learning as *N. pulcher* held in groups outperformed *T. temporalis* held in pairs (Experiments 3 and 4), whereas *N. pulcher* did not consistently outperform *T. temporalis* when both species were held in identical social conditions (Experiments 1 and 2). We also found that the social environment during testing and species’ sociality interact to affect task performance in a foraging task. *T. temporalis* outperformed *N. pulcher* in the truly solitary and most-social (conspecific always visible) conditions, but both species performed comparably in the intermediate social condition (conspecific visible only during trials), and *N. pulcher* outperformed *T. temporalis* in the social control condition (conspecific present but always invisible), warranting further investigation into the impact of sociality on learning ability. Use of more cognitively demanding tasks, such as reversal learning, could help further discriminate between differences across species’ cognitive task performances and might reveal clearer performance differences between species and individuals (Buechel et al., 2018). Our findings emphasise the importance of standardising social environments relative to the test species’ natural social landscape, as well as the need to minimise the distraction that extraneous conspecifics may provide during testing. Although we were not able to show that more-social cichlid species have a greater general capacity for learning when compared to less-social species, we have devised learning paradigms that can easily be applied to other cichlid species for further comparative experiments. Cichlids are an ideal group for further testing because they are a speciose group with many closely related species that have highly varied social lives despite living in rather similar ecological environments (Dey et al., 2017).
